# Essential Palatal Tremor Following an Upper Respiratory Tract Infection: A Case Report

**DOI:** 10.7759/cureus.12543

**Published:** 2021-01-07

**Authors:** Khalid Azam, Ayesha Kanwal, Manahil Chaudhry, Noreena Iqbal, Ayesha Malik

**Affiliations:** 1 Pulmonology, CMH Lahore Medical College and Institute of Dentistry, Lahore, PAK; 2 Medicine, CMH Lahore Medical College and Institute of Dentistry, Lahore, PAK; 3 Medicine, Hameed Latif Hospital, Lahore, PAK; 4 Medicine, Milton Keynes University Trust Hospital, Milton Keynes, GBR

**Keywords:** myoclonus, palatal myoclonus, palatal tremor, tinnitus

## Abstract

Bilateral objective tinnitus is a rare accompanying manifestation of an underlying palatal tremor (PT). PT can either be secondary to a lesion in the triangle of Guillain and Mollaret, or it can present without a causal organic lesion. In this report, we present an unusual case of a young female with objective tinnitus revealing a PT.

## Introduction

Tinnitus is a common otologic symptom and is defined as the conscious awareness of a sound in the absence of an external auditory stimulus [1]. It can either be objective or subjective, with objective tinnitus being a less common presentation. Objective tinnitus is when the sound is perceived both by the patient and the examiner, while subjective is when the sound is heard just by the patient. Palatal tremor (PT) is a rare cause of objective tinnitus.

PT, previously known as palatal myoclonus, is a rare movement disorder presenting as continuous rhythmic spasms of the palatal muscles. These involuntary contractions present as “clicking” sensations in the ears [2]. PT occurs primarily in young adults [3]. It is classified into two types: symptomatic and essential. Symptomatic PT is caused by a lesion in the triangle of Guillain and Mollaret (dentato-rubro-olivary pathway) and is associated with hypertrophic olivary degeneration visible on MRI of the brain, while essential PT has no identifiable cause [4]. Some other contrasting features between the two types include the following: the primary muscle involved in essential PT is the tensor veli palatini, while in symptomatic PT, it is the levator veli palatini; the ear clicking is more frequent in symptomatic PT, and while the clicking sound can disappear during sleep in essential PT, it is persistent in symptomatic PT [4].

## Case presentation

A 20-year-old female presented to our outpatient department complaining of intractable “clicking” tinnitus for the last two years. She had been in her usual state of health two years back when she had experienced an upper respiratory tract infection (URTI) for which she had taken antibiotics for one week, which had led to the resolution of the infection. One week after the resolution of her symptoms and discontinuation of antibiotics, she had developed clicking in both ears. She reported her symptoms to be persistent and bilateral (left > right). She denied any history of ongoing headache, blurring of vision, dysphagia, dysarthria, fever, weight loss, or any other neurological or auditory symptoms.

An otologic examination revealed normal tympanic membranes and hearing was normal on audiological testing (pure tone and impedance audiometry). There were continuous, involuntary rhythmic contractions of the soft palate (Video 1), and the physician could appreciate the clicking sounds, which were asynchronous with the pulse. The lips, tongue, larynx, and diaphragm were not involved. The rest of the examination was unremarkable.

**Video 1:** Objective tinnitus due to palatal tremor

She had no significant past medical or family history. The contractions persisted during phonation and sleep. Her tinnitus was refractory to treatment with various antidepressants, antiepileptics, skeletal muscle relaxants, and physiotherapy. She underwent an MRI of the brain, which revealed no notable lesions. She has been advised to undergo a trial of botulinum toxin therapy and is currently awaiting the procedure.

## Discussion

In this report, we presented a case of essential PT in a 20-year-old Asian female. The most recognized symptom to be reported with an underlying PT is tinnitus, often described by the patient as a clicking sound that is synchronous with the soft palate contractions when examined orally or nasendoscopically. Clicking tinnitus is a rare otologic symptom that has been attributed to various causes, such as a temporomandibular joint click, a patulous eustachian tube, middle ear myoclonus, and PT. Pulec and Simonton in 1961 proposed that the click sound is produced when there is a breaking of surface tension as the wall of the eustachian tube snaps open and shuts, mainly caused by a forceful contraction of the levator veli palatini or the tensor veli palatini [5].

East et al. [6] conducted a study (1987) on tinnitus-masking devices in which they recruited patients who had presentations similar to our patient, i.e., objective tinnitus with an essential PT. In another report, Schwartz et al. [7] described an eight-year-old boy who was voluntarily able to reproduce the clicking sounds secondary to an underlying PT as a learned behavior.

The objective tinnitus of our patient persisted during sleep, which is a feature of symptomatic PT, which is often neurological in origin. However, due to the absence of any recognizable organic cause, it was labeled as essential palatal myoclonus. Nonetheless, one plausible explanation was that the tinnitus of our patient proceeded from an undiagnosed URTI. Björk [8] has reported a similar case: a 33-year-old patient who started experiencing these clicking sounds after suffering from a severe influenza-like infection. However, an association between URTI and PT has not been established yet, and that is why we labeled it as essential, rather than symptomatic PT.

Various treatment options have been described for this condition. For essential PT, some of the recommended treatment options are anticonvulsants such as sodium valproate, carbamazepine, or clonazepam, and intra-lesional injections of therapeutic botulinum neuromuscular junction toxin into the insertion of the levator and/or tensor veli palatini muscles [7]. Conversely, no therapeutic regimes have proven to be satisfactory, including anxiolytics, antidepressants, and anticonvulsants, much like in our patient. On the flip side, botulinum toxin, owing to its selective muscle paralysis, is a front-line modality in the treatment of this condition and has been proven to achieve complete resolution of symptoms in four out of five patients [9].

## Conclusions

This case report highlights the phenomenon of PT occurring in a patient after she has experienced a URTI, thereby implying a possible association between the two conditions. PT, though rare, should be part of the differential when clinicians encounter cases of bilateral objective tinnitus so that a prompt diagnosis can be made and patients can be offered appropriate treatment.
